# Correction: Validation of the Polish version of the Brief Resilience Scale (BRS)

**DOI:** 10.1371/journal.pone.0244895

**Published:** 2020-12-30

**Authors:** The PLOS ONE Staff

There are errors in the author affiliations. The publisher apologizes for the errors. The correct affiliations are as follows:

Karol Konaszewski^1^, Małgorzata Niesiobędzka^1^, Janusz Surzykiewicz^2,3^

**1** Faculty of Education, University of Bialystok, Bialystok, Poland, **2** Faculty of Philosophy and Education, Katholische Universität Eichstätt-Ingolstadt, Germany, **3** Faculty of Education, Cardinal Stefan Wyszynski University in Warsaw, Poland.
