# Supplementary material for: Validation of the Polish version of the Brief Resilience Scale (BRS)
Source: PLoS One. 2020 Aug 10;15(8):e0237038. doi: 10.1371/journal.pone.0237038 (PMC7416919; doi:10.1371/journal.pone.0237038)
Supplement: S1 File — (PDF) [file pone.0237038.s001.pdf]

**Polish version of the Brief Resilience Scale (BRS)**

**Instrukcja**: Korzystając z poniższej skali zaznacz **zakreślając** jedną z cyfr, w jakim stopniu nie zgadasz się lub zgadzasz się z każdym z twierdzeniem.

1 = zdecydowanie nie zgadzam się

2 = nie zgadzam się

3 = ani nie zgadzam się, ani zgadzam

4 = zgadzam się

5 = zdecydowanie zgadzam się

| | | | | | |
|---|---|---|---|---|---|
| 1. Mam skłonność do szybkiego stawania na nogi po ciężkich czasach | 1 | 2 | 3 | 4 | 5 |
| 2. Jest mi trudno przetrwać stresujące wydarzenia | 1 | 2 | 3 | 4 | 5 |
| 3. Z łatwością dochodzę do siebie po stresujących wydarzeniach | 1 | 2 | 3 | 4 | 5 |
| 4. Trudno mi pozbierać się kiedy dzieje się coś złego | 1 | 2 | 3 | 4 | 5 |
| 5. Zazwyczaj przechodzę przez trudne sytuacje bez większych problemów | 1 | 2 | 3 | 4 | 5 |
| 6. Zwykle dużo czasu zajmuje mi przezwyciężanie niepowodzeń w życiu | 1 | 2 | 3 | 4 | 5 |
