# Supplementary material for: Validation of the Polish version of the Brief Resilience Scale (BRS)
Source: PLoS One. 2020 Aug 10;15(8):e0237038. doi: 10.1371/journal.pone.0237038 (PMC7416919; doi:10.1371/journal.pone.0237038)
Supplement: Attachment — Submitted filename: Response to Reviewers.docx [file pone.0237038.s002.docx]

Dear Frantisek Sudzina

Academic Editor

Thank you for your letters and the opportunity to revise our paper on ‘Validation of the Polish version of the Brief Resilience Scale (BRS)’. The suggestions offered by the reviewers have been immensely helpful, and we also appreciate your insightful comments on revising the paper.

I have included the reviewers comments immediately after this letter and responded to them individually, indicating exactly how we addressed each concern or problem and describing the changes we have made. The revisions have been approved by all authors and I have again been chosen as the corresponding author. The changes are marked in yellow in the paper as you requested, and the revised manuscript is attached. We also corrected the manuscript according to Journal Requirements.

We thank you for your continued interest in our research.

Sincerely,

**Answers:**

**Reviewer #1:**

Specifically, some of the aspects that deserve attention have to do with:

1. Introduction: On the one hand, authors should include more citations to support the different definitions they propose. Evidence that has no scientific support is made in the first paragraph of the introduction. The same happens in the third paragraph section that refers to "Researching abilities" the authors should complete with current references that accompany and give value to the statements they present. On the other hand, the authors should refer to the theory of coping strategies, being these relevant to understand the definition of resilience presented here. The authors could quote:

- Lazarus, R. S., y Folkman, S. (1984). Stress, Appraisal, and Coping. New York, NY: Springer.

- Cantero-García, M., & Alonso-Tapia, J. (2018). Coping and Resilience in Families With Children With Behavioral Problems. Revista de Psicodidáctica (English ed.), 23(2), 153-159.

- Alonso-Tapia, J., Rodríguez-Rey, R., Garrido-Hernansaiz, H., Ruiz, M., y Nieto, C. (2016). Coping assessment from the perspective of the person-situation interaction development and validation of the Situated Coping Questionnaire for Adults (SCQA). Psicothema, 28(4), 479-486. http://dx.doi.org/10.7334/psicothema2016:19

- Villasana, M., Alonso-Tapia, J., y Ruiz, M. (2016). A model for assessing coping and its relation to resilience in adolescence from the perspective of “person–situation interaction”. Personality and Individual Differences, 98, 250–256.

- http://dx.doi.org/10.1016/j.paid.2016.04.053

**Answer 1:** Thank you for suggestion devoted the scientific suport made in the introduction. We thoroughly read the suggested literature and completed references.

2. Methodology: Authors should delve further into sociodemographic characteristics.

**Answer 2:** We added sociodemographic characteristics in the study 3.

3. As well as clarifying why one works with such a wide age range. Furthermore, it would be advisable to expand the n of the study 2.

**Answer 3**: A wide age range was in the study 1 and 3, except for study 2. The study group only consisted of students aged between 20 and 29 and it was a convenient group. We agree that the size of the sample in study 2 should be extended what we suggested in the paragraph devoted limitations.

4. In addition, it is necessary to clarify how the data collection was carried out.

**Answer 4:** We added characteristic of CAWI method to clarify the data collection method in study 3.

5. The fit indexes of the model are very good, although it is not clear why the choice is made to separate the positive and negative items. Better justify the structural model.

# Answer 5: Thank you for suggestion, we added more information about the tested model in the study and tried to explain more comprehensively the motives underyling the choice of the structural model.

6. It is unclear whether the item scores were treated as categorical-ordered or continuous variables.

**Answer 6:**The item scores were treated as categorical-ordered variables.

7. The choice of estimation procedure should be justified.

**Answer 7:**Thank you for suggestion. We added information about ML procedure.

8. Discussion. You point out the methodological and theoretical limitations in a clearer way.

**Answer 8:** We added section „limitations”.

**Reviewer #2:**

1. The authors should systematize the name of the variable. Currently, two terms are used in the work: resilience and resiliency. Consistent terminology would allow for better understanding of the text.

**Answer 1:** We agree, it has been corrected.

2. My suggestions is that the "Data Analysis" section should not indicate hypotheses ("we expected..."). This section should focus primarily on the description of data analysis methods. Please consider describing the study objectives and hypotheses in a different place of the text – e.g. in the first paragraph describing the study.

**Answer2:** Thank you for suggestion, but we decided not to change the structure of the text.

3. The authors should translate foreign literature items into English.

**Answer 3:** Thank you for suggestion - it has been corrected.
